# Illuminating the Black Box: Interpreting Deep Neural Network Models for Psychiatric Research

**DOI:** 10.3389/fpsyt.2020.551299

**Published:** 2020-10-29

**Authors:** Yi-han Sheu

**Affiliations:** ^1^Psychiatric Neurodevelopmental and Genetics Unit, Department of Psychiatry, Massachusetts General Hospital, Boston, MA, United States; ^2^Department of Psychiatry, Harvard Medical School, Boston, MA, United States; ^3^The Stanley Center, Broad Institute of Harvard and Massachusetts Institute of Technology (MIT), Cambridge, MA, United States

**Keywords:** model interpretability, explainable AI, deep learning, deep neural networks, machine learning, psychiatry

## Abstract

Psychiatric research is often confronted with complex abstractions and dynamics that are not readily accessible or well-defined to our perception and measurements, making data-driven methods an appealing approach. Deep neural networks (DNNs) are capable of automatically learning abstractions in the data that can be entirely novel and have demonstrated superior performance over classical machine learning models across a range of tasks and, therefore, serve as a promising tool for making new discoveries in psychiatry. A key concern for the wider application of DNNs is their reputation as a “black box” approach—i.e., they are said to lack transparency or interpretability of how input data are transformed to model outputs. In fact, several existing and emerging tools are providing improvements in interpretability. However, most reviews of interpretability for DNNs focus on theoretical and/or engineering perspectives. This article reviews approaches to DNN interpretability issues that may be relevant to their application in psychiatric research and practice. It describes a framework for understanding these methods, reviews the conceptual basis of specific methods and their potential limitations, and discusses prospects for their implementation and future directions.

## Introduction

Psychiatric disorders are common and a leading cause of disability worldwide. Substantial research has been done in the field, but major questions about their causes, treatment, prediction, and prevention remain unanswered. In part because mental phenomena and their disorders are inherently multidimensional and reflect complex dynamic processes, psychiatric research comprises a unique set of challenges that have not been tractable to date using conventional approaches. The validity of psychiatric constructs and their measurements and the interplay between and within bio-psycho-social factors to determinants might not be readily describable by heuristic knowledge or by simple models of dynamics currently established. Despite tremendous efforts, overall progress in understanding and treating psychiatric illnesses has been modest in the past decades.

The emergence of Big Data and recent developments in machine learning (ML) might provide a venue to tackle some of the challenges. Deep neural networks (DNNs) (1, 2), a specific type of ML model, could be particularly helpful in some cases. DNN models are inspired by biological brains, using artificial neurons (e.g., mathematical analog to biological neurons) as units and, with those, building a network by wiring a large number of units together in specific ways. Two unique theoretical properties make them particularly appealing to psychiatric research, namely the capability of finding and mapping more complex patterns in data compared to other models, and the ability to automatically learn important and, at times, novel aspects of information through sequential data transformations (e.g., “representation learning”). Empirically, in the field of healthcare, they have already achieved groundbreaking progress in various applications: for example, drug discovery (3), protein folding (4), and clinical risk prediction (5). There is also published work on using representation learning to potentially enhance the validity of psychiatric taxonomy (6).

However, it is also known that DNNs possess a set of lingering issues that remain to be improved. For example, there is increasing awareness of the challenge of model interpretability (7–15). Complex ML models, such as DNNs, are sometimes referred to as “black box” models because their mechanisms of making decisions are not explicitly accessible to human cognition. In the context of psychiatric research, model interpretability is desirable for the following reasons: (1) for clinical applications, building trust between the model and stakeholders is fundamental for adoption of the tool. Trust is directly related to the level of understanding of the inner working of the model (e.g., “knowing why”). It is known that DNNs are capable of making accurate predictions based on “peripheral” features or noise but that contain no heuristic or scientific meaning other than statistical correlation with the labels. In this context, the models could be more vulnerable to adversarial attacks and noise when applied to out-of-distribution data (16, 17). For example, one can build a well-performing classifier for diagnosis of depression based on internal data that is, in fact, less reliable when applied to real-world data. With appropriate model interpretation, researchers and clinicians can make better judgments about whether the model is trustworthy in a given scenario, supported by their expert knowledge. (2) Model interpretation helps to identify critical aspects of the data (e.g., the underlying biological mechanism as shown in neuroimaging tools) and could help the progress of science in both better understanding the subject matter and improving the model. (3) For psychiatry in particular, a significant proportion of clinical decisions are made by jointly considering objective conditions and subjective considerations—for example, preferences to choose over a certain medication side effect profile vs. another, etc. Knowledge about how the model makes decisions allows the flexibility to adjust to additional human preferences and value judgments not readily incorporated in each instance. (4) Finally, on the legal side, model interpretability is explicitly stated as a requirement by the General Data Protection Regulation set by the European Union (18).

**Figure 1:**
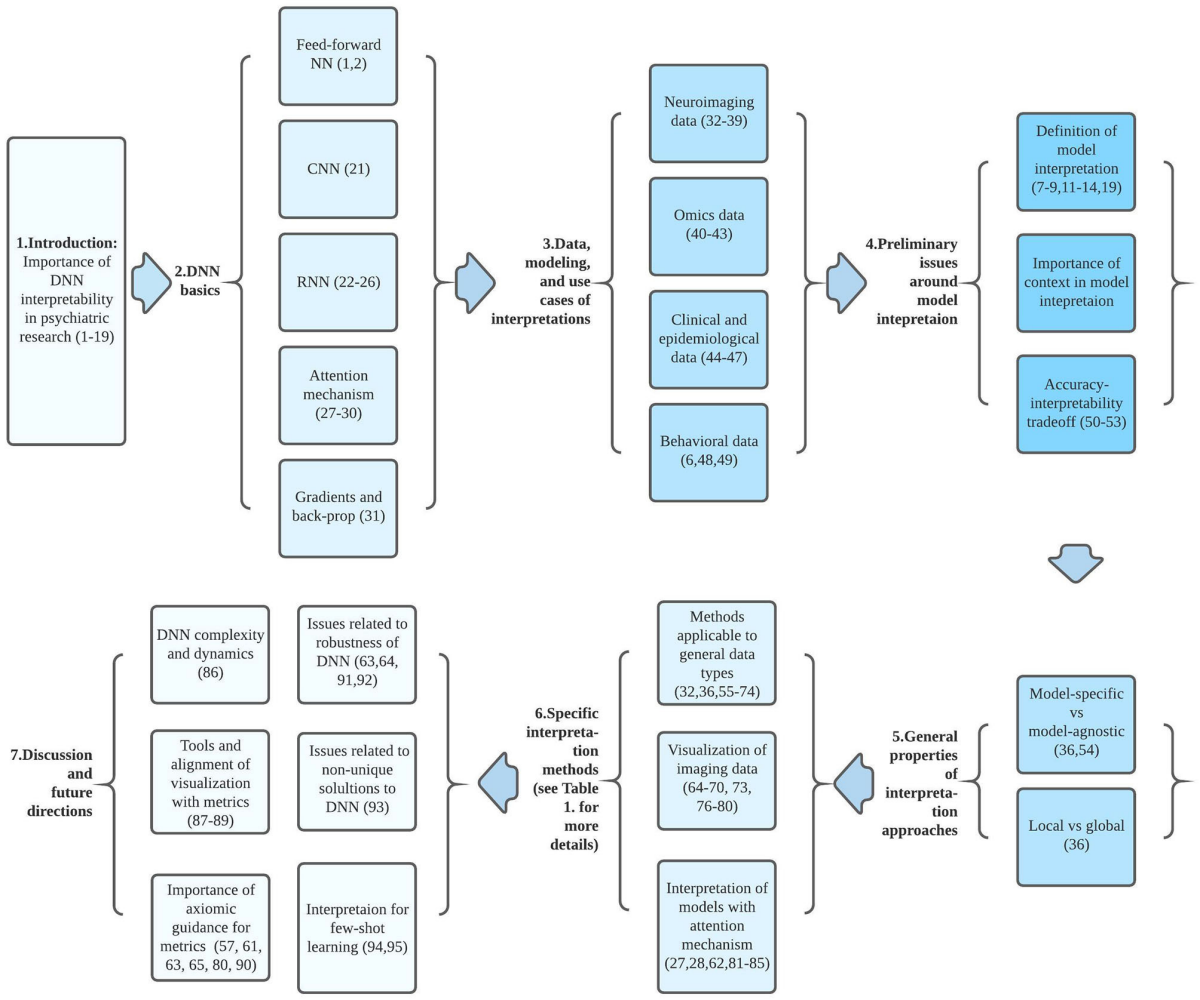
Conceptual flow chart connecting ideas and articles reviewed and discussed in this paper. Each block between a set of arrows corresponds to a particular section of the paper. Numbers in parenthesis indicate relevant referenced articles.

Efforts to improve the interpretability of complex ML models has been an active area of research, and several recent reviews have addressed recent developments in this area for an ML audience (7–11, 13–15). In this article, we aim to summarize some of these issues in the context of their potential application to psychiatric research. Starting from a brief introductory sketch of DNNs, we then discuss general considerations regarding DNN interpretation methods; the current status of available interpretation methods; and their limitations, implementations, and possible future directions. Our main goal is not to provide an exhaustive review, but to introduce basic principles and emerging approaches to DNN interpretability that may provide context for psychiatric researchers interested in applying these methods. On the other hand, ML researchers interested in mental health research might also find this article helpful. In this paper, we discuss interpretability for supervised learning as most interpretation methods were developed under this context, but many of the methods can be generalized to semisupervised learning as well. Figure 1 may serve as a guide to aid readers in navigating the conceptual flow of this paper.

## DNNs in a Nutshell

### Basics of DNNs

DNNs belong to the broader class of neural networks (NNs) (1, 2). As mentioned, the basic unit of an NN is an artificial neuron, which is a simple simulation of biological neurons. Biological neurons take input from other neurons, form an action potential, and then output signals to subsequent neurons via synapses. Artificial neurons are also connected in an analogous way, and synaptic strengths are designated by numeric weights with higher weights indicating a stronger connection. Action potential is simulated by a nonlinear “activation function,” which typically has a drastic output value change after input value exceeds a certain threshold. NNs are typically composed of “layers” of artificial neurons, which take a signal from their counterparts in the preceding layer and output to the next after the aforementioned transformation although, typically, there is no connection between neurons in the same layer. In real-world applications, the number of neurons in each layer is usually large (starting from the order of hundreds to tens of thousands depending on the design). To further link NNs to other statistical models, it is noteworthy that logistic regression can be expressed as a simple case of NN—it is a, NN with only two layers (input and output) with all inputs linked to a single output cell and applying a logistic activation function at the output cell.

A DNN is a specific case of the general class of NNs such that it has at least three layers in its structure: an input layer, an output layer, and at least one layer in between, designated as a “hidden” layer. What makes DNNs unique are the hidden layers; because each layer includes a step of linear and nonlinear transformation, hidden layers make DNNs “compositional” in nature (e.g., functions of functions), which is shown to greatly increase the patterns that can be expressed by the model (19). On the other hand, it is in part the existence of these hidden layers in NNs that has contributed to concerns about their interpretability.

### Common DNN Architectures

**Figure 2:**
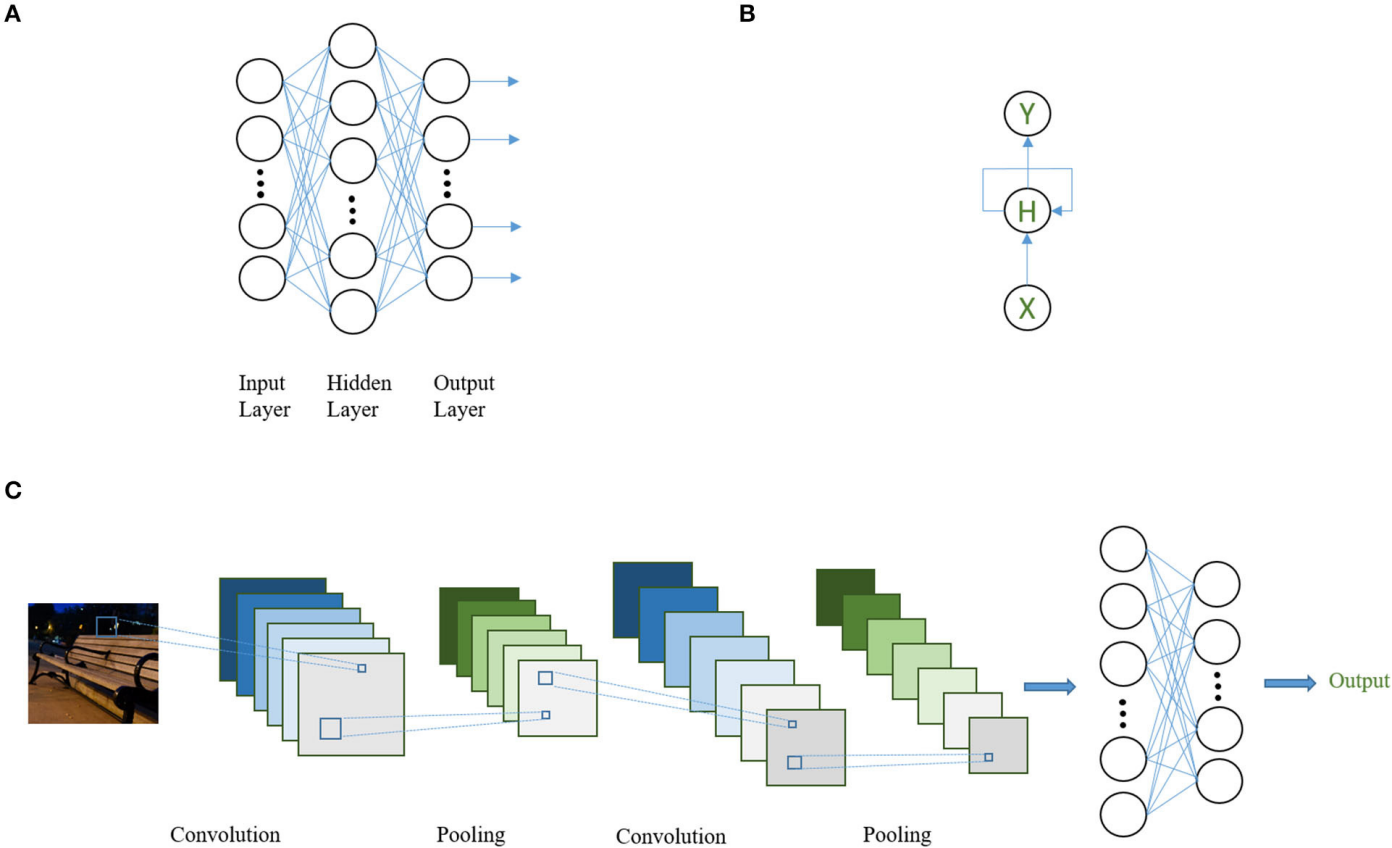
Schematic diagram for three common DNN architectures. **(A)** A three-layer, feed-forward NN (with one hidden layer). Each circle represents one artificial neuron. All neurons in one layer are connected to all neurons in its adjacent layer but not to the neurons in the same layer. **(B)** A simple RNN. Here, each circle represents a layer of neurons. Information from the hidden layer of the previous step is allowed to enter the following step. X: input layer; H: hidden layer; Y: output layer. **(C)** A convolutional NN with two convolutional layers, two pooling layers, and two feed-forward layers. At the first step, the convolutional filter transforms the input image into six “feature maps” (e.g., image transformed by the filter). The feature maps are then summarized by pooling, which reduces the dimension of the feature map, usually by taking the maximum values of smaller regions (i.e., 3 × 3) that covers the whole feature map and combining them with spatial relations pertained to produce a new feature map. This procedure is repeated two times and then the network is connected to a two layer feed-forward network to derive the final output.

Currently, there are three common types of DNN architectures, namely (1) feed-forward NN, (2) convolutional neural networks (CNNs) (20), and (3) recurrent neural networks (RNNs) (21) (Figure 2). These structures can be used as building blocks (in the form of layers) for a more complicated DNN in flexible ways as long as model training is computationally feasible. Feed-forward NNs are the basic type of DNNs in which the batch of neurons within each layer is connected to and only to those in the previous and the following layers. Information is propagated from the input layer through a sequence of hidden layers to the output in a straightforward fashion.

CNNs are motivated by imitation of biological vision systems and have been widely adopted for (but not limited to) computer vision and image-related tasks, such as reading pathology slides or brain images. The aim is to simulate the hierarchical nature of neurons in the vision cortex. The input neurons, usually representing pixels of an image, are typically connected to a smaller group of neurons that act as “filters,” which scan through the entire image. The filter processes through the entire input image by moving one or several pixels at a time, which, in the end, outputs a filter-transformed version of the image. The sliding nature of the scan is why the term “convolution” was coined as it operates analogously to the convolution operation in mathematics. The main characteristic of the CNN is the “weight sharing” of the filter in which the parameters defining the filter are fixed throughout scanning of the whole image. Filters are capable of learning to recognize meaningful abstracts (e.g., representations) of the image that are directly correlated with the task at hand. For example, to identify a table, representations such as sharp edges or a flat surface might be captured automatically.

An RNN is motivated by the fact that there are forms of data in which observations may not be independent, such as time sequential or text data. An RNN by design takes into account dependencies over the sequence by taking in inputs sequentially and allowing information contained in the hidden layer of the previous step to enter that of the following. Variants of basic RNNs, such as long-short term memory (LSTM) (22) and gated recurrent unit (GRU) (23), differ in the way information is allowed to propagate over time. These are motivated by addressing known issues in vanilla RNNs when transmitting information across a larger number of time steps and have shown actual improved performance in various tasks (24, 25).

### Attention Mechanism

Having discussed the three basic architectures of NNs, we turn our attention to computational processes that have developed to further improve learning by DNNs. An “attention mechanism” was first described by Bahdanau et al. (26) as a novel component to a DNN-based machine translation model [which is a type of task in natural language processing (NLP)]. Its idea is to incorporate the learning, for each input example, on which part of the input information the model should put emphasis, in the form of weights applied to specific inputs (e.g., the larger the weight, the more emphasis) into the DNN model. Implementing an attention mechanism with DNNs of various types provides powerful model improvements and can yield state-of-the-art performance across many NLP tasks (27). In fact, application of an attention mechanism is not confined to modeling texts, but rather natural to any DNN model assuming a sequential data structure. Attention mechanisms have also been adopted for modeling imaging data although they have not been as prevalent as they are for sequential data (28, 29). Because an attention mechanism is an indicator of importance, it could provide a venue to understanding model decisions, which are discussed in a later section.

### Learning Model Parameters—Gradients and Back-Propagation

In simpler models, such as linear regression, model parameters can sometimes be estimated with a closed form, fixed solution. For complex ML models, the parameters are usually difficult to solve directly, and one would have to rely on numerical approximations to obtain their estimates. An important class of these methods is based on updating the parameter to be learned with “gradients” during model training. In a heuristic sense, gradients express how model behavior would change with respect to a small change in a certain parameter or input value (e.g., the respective partial derivative) at the given value. Gradients are calculated for DNNs for all parameters using a technique called “back-propagation” (30), which is essentially applying a chain rule in calculus to derive derivatives with regard to the objective function (e.g., the function on which the model is being optimized) for every parameter in the model, taking advantage of the compositional nature of DNNs. Since gradient values corresponds to how model output would change by a subtle change in the input, they can also be used as a means for model interpretation.

## Data Types, DNN Modeling, Utilities, and Examples of Model Interpretation in Psychiatric Research

The scope of psychiatric research is massive and involves a variety of data types and structures, upon which model interpretation also depends. For this reason, we briefly discuss the structure of commonly seen data types in psychiatric research, how to model them efficiently by DNNs for a given research question, and why model interpretation could be beneficial. It is important to note that DNNs are inherently flexible, and there is no presumption of a “definitive” way to fit a particular type of data. In many cases, data can be fit well with more than one method or a mixture of methods.

### Neuroimaging Data

Electroencephalogram (EEG) (31, 32), event-related potential (ERP) (33), magnetic resonance imaging (MRI) (34), functional magnetic resonance imaging (fMRI) (35), and positron emission tomography (PET) (36) are imaging tools that are commonly used in psychiatric research. These techniques generate images that are either static (MRI) or time-varying (EEG, ERP, fMRI, PET). Static images are mostly suitable to be fit with CNN-based models (37). When time is taken into account, the time series can either be modeled with a CNN (31) or a mixture of CNN and RNN (35). In the case of neuroimaging, interpretations of the DNN models applied could shed light on the underlying brain structure or mechanism that corresponds to a measured phenotype or any other metrics of interest. An example is the EEG classifier and its interpretation described in Ke et al. (31).

### Omics and Molecular-Level Data

Genetics and other—omics data are important in psychiatric studies as most psychiatric disorders are at least partially heritable (38, 39). Although genetic coding information is sequential by physical structure, the exact dynamics of interplay between genetic and other molecular-level information is largely undetermined, less the typical structure of the DNA-RNA-protein cascade. Therefore, in the context of functional genomics, one might be inclined to model such assuming the least on data structure, such as a deep feed-forward network. For example, in Wang et al. (40), the authors employ a model based on the deep Boltzmann machine (41), the probabilistic analogue of feed-forward NN, to classify cases versus noncases for several psychiatric disorders using integrated—omics data. In this particular work, the authors derive model interpretation to a certain degree by assigning the hidden nodes to inherit heuristic meanings from observed nodes using a defined rule to construct linkage pathways between genotypes and phenotypes. More sophisticated model interpretation methods would allow additional biological insight to be drawn, for example, the contribution of a particular gene to the phenotype of interest in a certain cell type.

### Clinical and Epidemiological Data

Clinical and epidemiological data range from those focusing more on individuals (i.e., more in-depth data collected from relatively fewer subjects)—such as interview records in the forms of text of videos or comprehensive questionnaires—to those that collect data from a larger group of subjects but possibly less in-depth per subject, such as electronic health records (EHR), health insurance claims databases, and cohort data (42–45). In most cases, these data are heterogeneous in structure. For example, EHR can contain quantified as well as text data. An optimal choice of model class depends on the actual data type involved and the study question. For example, if one is analyzing text scripts, then a model with an attention mechanism might be appropriate. If one is building a risk-prediction model for a certain phenotype, a feed-forward NN might be plausible due to minimization of prior assumptions to the data. On the other hand, derivation of model interpretation could be particularly crucial for models that are developed for clinical applications (e.g., decision support system) given the natural tendency for one to learn the basis for decisions made, not to mention those involving medical considerations that might involve risks and benefits. For example, one might build a suicide risk–prediction model to stratify those with higher or lower risk. Both the clinician and patient would then be inclined to be informed why, in a particular situation, the patient was classified as such.

### Behavioral Data

Broadly speaking, any data that reflect human behaviors would be potentially informative to psychiatric research. For example, data collected from mobile phones and Facebook use may provide clues to depression and anxiety (46, 47). In Dezfouli et al. (6), data collected through a bandit task is used to classify patients with bipolar disorder versus controls. Video or audio recordings of patients may also be used for modeling tasks, such as phenotyping. Again, the optimal model structure and interpretation method depend on the specific data collected. Interpretation of these models could facilitate a better understanding of the roles of behavioral features relative to the phenotype of interest.

## Preliminary Issues Around Model Interpretability

In this section, we briefly discuss some of the background issues of which to be aware around model interpretability.

### “Interpretability” Is Not a Precisely Defined Term

One of the recurring themes in the ML interpretability literature is the constant efforts toward a universally accepted definition of the term “interpretability” (8, 13, 14). Despite efforts (7–14), thus far, there has not been an established consensus as how interpretability should be best defined in the context of ML. In our view, the question of interpretability can be approached from two different perspectives: (1) the perspective of science, in which a precise, formulated definition is required, and (2) the perspective of the interpreter, which arises from the psychological need to construct meaning out of things. Although most previous works discuss the issue from the former (7–14), we begin our discussion with the latter. In this paper, we define interpretability as the capability of a subject matter to be faithfully translated into a language available and a meaning sensible to the interpreter. By “faithfully,” we emphasize alignment with science despite the definition being human-centered. Last, we avoid using commonly used synonyms of “interpretability” to minimize confusion.

### The Importance of Context in Interpretation

Discussions around model interpretability are often focused on its mathematical aspects. When applied to a specific task, however, it requires an added step of translating mathematical model components to the actual substantives. There is a substantial amount of contextual subtlety in psychiatry that cannot be readily extracted from quantified data, making this step particularly critical for psychiatric researchers. Thus, it is important for them to work closely with ML specialists to start from the design phase of a model and make valid and meaningful interpretations that naturally align with the contextual need.

### The General Accuracy–Interpretability Trade-Off

DNNs are not alone in being tagged the “black box” property. Model classes that are usually deemed “interpretable” mathematically can become opaque when translated into context as model size grows. For example, it is reasonable to state a linear regression model containing fewer terms to be easily interpretable. However, it is less clear if a linear regression with thousands of dimensions and a collection of higher order interaction terms can be called interpretable; the model parameters would retain the same interpretation, but constructing a heuristic explanation from the subject matter becomes hard. The same applies to single decision trees when the tree grows deeper.

Ensemble tree-based ML models, such as random forests (48) and gradient boosting (49), as well as support vector machines (50) and their variants are among the best performing non-DNN ML models by accuracy metrics. However, they also are less directly interpretable compared to decision trees and logistic regression, and methods were developed to help better understand how these models make decisions (51). As discussed in later sections, many of these methods can be applied to any ML models (i.e., model-agnostic) and, thus, can be applied to DNNs.

Interpreting DNNs comes with a unique set of challenges. DNNs, unlike other models, consist of hidden layers in which automatic feature learning occurs, and one would be inclined to know the actual workings (i.e., the transformations taking place and the correlation between arbitrary layers) in these nodes in addition to the relationship between inputs and outputs. Also, as introduced previously, the structure of DNNs can vary, and the delicate information flow (i.e., via gradients, weights, and transformations) make them intrinsically a more complex subject to study. That said, there do exist tools to help us understand their mechanisms to an extent as we discuss in the following sections.

## General Properties of Approaches for DNN Interpretations

Before we introduce specific methods, we first categorize them into a structure consisting of two important dimensions as previously described (51): (1) the classes of models to which the method is applicable (i.e., model-specific vs. model-agnostic) and (2) the scope of data at which the method looks (i.e., local vs. global).

### Model-Specific vs. Model-Agnostic Methods

Model-specificity means that the interpretation method at hand can only be applied to a certain class of models. On the other hand, model-agnostic methods are applicable to any ML models in general. Model interpretation is carried out by inspecting components of a given model. Some components are universal to all models (i.e., inputs and outputs), and some are specific to certain structures of models; the same applies to the corresponding interpretation methods. For example, feature importance for tree-based methods—such as random forest or gradient boosting—are calculated from the number of split nodes involved for each feature and can only be carried out to models with the corresponding structure (52). DNNs are unique in ways that they are structurally compositional, followed by delicate calculations in gradients, and sometimes incorporate an attention mechanism. Accordingly, methods utilizing these structures would then be specific. In contrast, methods that involve direct manipulation of common structures, such as model inputs, are generally model-agnostic.

### Local vs. Global Interpretations

An interpretation method can provide either summarized information about model behavior for each respective feature regardless of its value (i.e., global) or information about model behavior around the neighborhood of a specific data point (i.e., local, which may be data for a single patient or a single image). The decision between global vs. local interpretations needs to be made with respect to the context of the application. For example, the former might be more suitable when a model is applied to determine the strength of a relationship between a certain predictor and population-level (e.g., aggregated) outcomes, and the latter might be preferable when the goal is to inform rationales for modeled decision making for a specific patient. Many of the DNN-specific interpretation methods are local because most DNN-specific components behave differently in accordance with their value at model evaluation.

## Specific Interpretation Methods

Table 1 summarizes selected interpretation methods and their characteristics along the axes of locality and specificity to models. In psychiatry, researchers may be working with—omics/molecular data, cohort or EHR data, free text, imaging or magnetic/electrophysiological data, behavioral records, questionnaires, or time series signals. To bridge this interest from psychiatry in interpretation of their use of DNN models, we introduce specific interpretation methods in three categories, namely (1) methods applicable to data of any type (hereafter referred to as “general inputs”), (2) visualization techniques for medical imaging data, and (3) utilizing an attention mechanism for model interpretation with free text data (26).

**Table 1:** Summary of approaches to DNN interpretation.

**Approach**	**Model Applicable**	**Scope of Input**	**Properties**	
**General methods**				
Permutation importance (48, 52)	Model agnostic	Global	**Idea**	Permute values within each predictor and calculate score based on performance drop
			**Footnote**	Results may be biased if predictor of interest is correlated with other predictors (53)
Partial Dependence Plot (PDP) (49)	Model agnostic	Global	**Idea**	Plotting values of predictor of interest versus outcome with all other predictors averaged out
			**Footnote**	May be biased when predictors are correlated; difficult to visualize when number of interested predictors is large (54, 55)
Individual Conditional Expectation (ICE) (56)	Model agnostic	Global	**Idea**	Similar to PDP, but plotted for individual examples
			**Footnote**	May be biased when predictors are correlated; difficult to visualize when number of interested predictors is large (51, 54)
Local Interpretable Model-agnostic Explanations (LIME) (57)	Model agnostic	Local	**Idea**	Approximates model locally with another interpretable model and data representation
			**Footnote**	The procedure of finding the neighboring sample points may result in unrealistic data point; results may not be robust (51, 58)
Deep Learning Important FeaTures (DeepLIFT) (59)	DNN specific	Local	**Idea**	Computes average gradients at the input value of interest versus a reference value. Calculation facilitated by the compositional DNN structure
			**Footnote**	Results may be inaccurate in the presence of multiplicative interactions between predictors (60), robustness issues (58)
Shapley Additive Explanations (SHAP) (61)	Model agnostic or DNN-specific	Local	**Idea**	Calculates Shapley values through various approaches for interpretations using linear additive models, such as LIME and DeepLIFT
			**Footnote**	Robustness issues (58)
**Methods primarily coupled with saliency maps and imaging data**				
Perturbation-based methods (62–65)	Model agnostic or DNN/CNN specific	Local	**Idea**	Perturbs input values of a specific example and observes the change in modeled prediction
			**Footnote**	Computationally expensive (66)
Gradient-based methods (62, 67–72)	Mostly DNN/CNN specific	Local	**Idea**	Calculates score for each feature at the input of interest based on the gradient values with respect to modeled prediction
			**Footnote**	Meaning of the interpretation itself is unclear. Some methods have shown insensitivity to data or weight permutations (60)
**Methods primarily coupled with attention mechanism and text data**				
Attention weight visualization (73)	DNN models with attention mechanism	Local	**Idea**	Visualizes attention weight by showing heat map with corresponding text
			**Footnote**	Intuitive, but attention weights may not be totally causal to model decisions (74)
Attention saliency (75)	DNN models with attention mechanism	Local	**Idea**	Visualize scores based on absolute value of the derivative of the model output with respect of the unnormalized attention weight
			**Footnote**	Properties not yet investigated in depth
Word token analysis (76)	DNN models with attention mechanism;	Local	**Idea**	Analyzes spatial relationships of tokens transformed across attention layers with dimension reduction
	Position-preserving models		**Footnote**	Works only with model architectures with positional alignment between input and output sequences

### Interpretation Methods Applicable to General Inputs

Interpretation methods under this class utilize model components common to all DNNs, thus making them applicable in most research contexts. For example, one might be interested in building a risk-prediction model for a disorder of interest with a mixture of different data types (e.g., quantified clinical measurements, text data, imaging data, or genetic data) as predictors. The following methods allow interpretation of each predictor regardless of its data type.

#### Permutation Feature Importance Scores

Permutation feature importance score is a model-agnostic and global method (48, 77). The idea is to permute values of each predictor one at a time and evaluate performance metrics of models in which values of each predictor are permuted against those of the model in which the original input is used. Predictors contributing to a larger drop in model performance are given a higher importance score.

In the case of DNNs, it is preferable to retrieve permutation importance scores from test data (instead of training data). The first reason is due to computing time; to run on training data would require retraining the model the number of times equal to the number of features, which is, in some cases, not computationally feasible. A second reason is that researchers are generally interested in generalizing the model to data outside of training. Given that DNNs tend to over-fit during training, the interpretation methods that rely on importance scores might just capture noise that contributed to over-fitting (51).

An issue permutation importance scores possess and share with other methods involving singling out a particular predictor and then either performing shuffling or extrapolation on that predictor is when the predictor of interest is correlated with other predictors, which would result in making inferences with unrealistic data points or biased results (53). Another issue, as discussed in (59), is that permutation methods may underestimate the importance of features that have saturated their contribution to the output.

#### Partial Dependence Plot (PDP)

A PDP (49) is a model-agnostic and global interpretation method. It intuitively plots one or two predictors of interest on one axis and the output on the other axis, averaging out the effects of other predictors over their respective marginal distributions.

Despite its simplicity, PDP is also known to produce biased results when predictors are correlated; it represents a commonly violated assumption of pair-wise independence among predictors (54). It also becomes increasingly difficult to visualize information with a large number of predictors (55).

#### Individual Conditional Expectation (ICE)

ICE plots the predictor of interest against the outcome in the same way that PDP does (56). However, it differs from PDP in that ICE plots a graph for each example while holding all other predictors constant at their observed values.

Although ICE gives more detailed information on interactions between predictors than PDP (56), because configurations of other predictors are not collapsed to average values, it is similarly prone to bias when predictors are correlated; the plot may end up in regions where the combination of input values are improbable in such cases (51, 54).

#### Local Interpretable Model-Agnostic Explanations (LIME)

LIME is a model-agnostic and local interpretation method (57), which produces interpretations for specific examples. Local behavior of complex functions can be reasonably approximated by a simpler function, such as the first- or second-order approximation. In the same vein, LIME approximates the actual prediction model locally by training a model that is deemed interpretable (i.e., a linear model). The LIME procedure first converts the input data from its original form into a set of “interpretable representations.” Using text data as an example, in current practice, one might first transform “word embeddings” (78), which are vectorized representations of words and by itself incomprehensible to humans, to binary indictors for whether or not a particular word is present. Then, LIME constructs a “neighborhood data set,” which includes the example of interest, and a number of data points sampled close to that specific example in the interpretable representation space. After sampling, the neighborhood data are converted back to original features and run through the model to be explained, which, in turn, generates prediction for these inputs. The explanation model is then trained supervised on the labels generated by the actual prediction model using the interpretable representations as predictors. It is trained based on optimizing metrics that would encourage (1) closeness between the results coming from the explanatory model and the actual prediction model and (2) simplicity of the explanation model. Neighboring data points closer to the actual example of interest are given higher weights.

Although LIME is conceptually intuitive, two general issues should be considered: (1) The procedure of finding the neighboring sample points in LIME are defined arbitrarily, and the generated neighborhood data set may include data points that would rarely occur in real-world settings and are also at risk of over-weighting them (51). (2) It has been shown that LIME explanations may not be robust when attempting to explain nonlinear models (58). For example, in DNNs, attributions of predictors can vary significantly for neighboring data points, which is unfavorable. Therefore, although the idea of LIME is appealing, there are questions waiting to be solved, and researchers should remain cautious when applying this method.

#### Gradient-Based Methods

Gradient-based methods are mostly DNN-specific and local (62, 67–72, 79, 80). They take advantage of the fact that the compositional nature of DNNs allows the use of back-propagation (mentioned in section DNNs in a nutshell), which enables efficient calculations of the gradients. Intuitively speaking, the greater the gradient for a predictor, the more important it is for model output at the input value of interest. Because the gradient for a certain predictor varies across values and usually interacts with other predictors, these methods provide local explanations. Also, because gradients are calculated along the path of the whole DNN model, gradient-based methods can be applied between any two layers of the model.

In this class, integrated gradients and its variant (68, 80) is based on integrating gradients on a linear path from a reference value, chosen by prior knowledge, to the actual value of a predictor of interest for a certain data point. For example, for an imaging data set, the reference value could be zeroes for each color channel for each pixel. This method avoids the pitfall of using vanilla gradients such that it avoids assigning zero attribution to a predictor when the gradient at that data point is zero, but the output does, in fact, change when the value of this predictor changes from reference to the actual value (68).

Although most gradient-based methods are theoretically applicable to any DNNs, most of these were originally developed for imaging data to visualize locations in images important for modeling. Some gradient-based methods are specific to model CNNs, which are widely adopted for imaging data modeling. Visualization methods used for imaging data (“saliency maps”) are discussed in a later section.

#### Deep Learning Important FeaTures (DeepLIFT)

DeepLIFT is a DNN-specific and local interpretation method (59). Like integrated gradients (68), DeepLIFT attributes the importance of each predictor by comparing the model prediction using an actual predictor value to a reference value. However, instead of actually integrating gradient values within the range of interest, DeepLIFT defines “multipliers” as its base building block, which is a simple averaged corresponding change in the output by changing the input of interest from a reference value to the actual value of the data point in question (the “contribution”) and, therefore, can be perceived as a fast approximation to integrated gradients (59).

The overarching guidance of DeepLIFT is that the contributions of all predictors are linearly added to give the total change in the output [e.g., the “summation-to-delta” (59)]. Analogous to gradients in DNNs, the multipliers follow a chain rule–like property, analogous to that in calculus and back-propagation. With an attempt to preserve the summation-to-delta property, total contribution is allocated to each input and then further separated into positive and negative compartments within input. This way, DeepLIFT avoids misinterpretations that might arise from cancellation of numeric values with different signs. Note that, because the multipliers possess a chain rule–like property, DeepLIFT can assess contributions between any two arbitrary layers of neurons as other gradient-based methods can.

It is not recommended to use DeepLIFT in models (60) in which multiplicative interactions occur because it loses the summation-to-delta property [i.e., LSTMs (22)]. As a heuristic explanation, problems may arise in cases in which approximating gradients across a range of input using its average value is inappropriate.

#### SHapley Additive Explanations (SHAP)

SHAP (61) is a local interpretation method. It can be either model- or model-specific, depending on which variation is being used. It extends from Shapely values from cooperative game theory (51). A Shapely value is by itself a metric to calculate feature attribution. The idea of Shapely values is that all features “cooperate” to produce model prediction. In its classical form, the Shapely value is calculated as the weighted average of a change in modeled prediction comparing a model with and without a given predictor across all possible configurations (presence or absence) of other predictors. Because this approach requires repeated assessment of model performance for a large number of iterations, it is computationally intensive and infeasible for DNNs.

The SHAP framework starts with the observation that many of the feature attribution methods (e.g., LIME and DeepLIFT) can be categorized under a common class of “additive feature attribution models” for model interpretation. Then, within this additive model class, a unique solution to the explanatory model—the one that uses Shapley values as their coefficients to generate interpretations—would satisfy a set of favorable mathematical properties, such as accuracy in approximations (61). SHAP then introduces several efficient methods to obtain the Shapley value solutions (in contrast to the classical approach mentioned in the above paragraph) to additive feature attribution models. For example, Kernal SHAP is a model-agnostic method combining LIME and Shapely values; Deep SHAP (61, 81) is a DNN-specific method combining DeepLIFT and Shapely values, making use of the compositional nature of DNNs to improve computation efficiency to obtain Shapely value approximations.

Compared to general cases of LIME and DeepLIFT, SHAP interpretations provide an additional theoretical guarantee of several favorable properties, grounded by established proofs originating from game theory (61). However, as noted in Alvarez-Melis et al. (58), SHAP may also be vulnerable to the nonrobustness problem as observed in LIME.

### Visualization of Imaging Data

It is natural to use visualization techniques to make sense of imaging data. In psychiatry, these data are primarily generated through neuroimaging techniques described in section “Interpretability” is not a precisely defined term. As a hypothetical example for the utility of model visualization tools, say a researcher is interested in building a classifier for schizophrenia based on a set of imaging data, such as fMRI scans. Once the classifier is built, these techniques allow the researcher to highlight which particular areas of an image would be primarily responsible for a case-control classification.

One way of visualizing areas in an image crucial for the DNN model to make decisions is via a “saliency map” (71). Based on the approach to construct importance of predictors (pixels, etc.), saliency maps can be categorized by their underlying mechanism: (1) perturbation-based (62–65) or (2) gradient-based (62, 67–72) as discussed earlier. These methods are local and mostly DNN-specific.

The main idea of the perturbation-based approach is to remove or occlude a particular part of the input and observe the change of modeling prediction. Methods within this class differ in how the optimal areas to be perturbed are chosen and how the “change” in model prediction is assessed. One advantage of this approach is that we are then measuring the actual change of model output by intervening on the input of interest, instead of merely measuring the association between the output and input (62). However, due to the greater quantity of computation needed to implement this approach, in most cases, these require longer computation time (66) and are less widely used in the literature compared to gradient-based methods.

As mentioned, numerous gradient-based methods have been originally proposed to create saliency maps. These explanation methods include using original vanilla gradients for explanation [referred to as “vanilla” gradients (71)], guided back-propagation (69), deconvolutional networks (79), input × gradient (72), integrated gradients (68), grad-CAM (67), and guided grad-CAM (67). We can calculate the gradient for each feature (e.g., pixel) as the importance measure locally by using vanilla gradients for explanation. Deconvolutional networks and guided back-propagation differs from vanilla gradients in the way the nonlinear transformation was performed. Gradient X input calculates score based on the product of the gradient and the value of the feature. Grad-CAM is specific to models comprising a convolutional layer (CNN) and produces a feature importance heat map based on the product between the global average of gradients and the values of each feature for each channel of the convolutional layer of interest. Guided grad-CAM combines guided back-propagation and grad-CAM to enhance the spatial resolution from the original grad-CAM.

Given the popularity and plethora of saliency maps, Ancoma et al. (60) investigated whether or not these methods satisfy two criteria: (1) sensitivity to model parameter randomization and (2) sensitivity to data randomization. In this context, sensitivity means whether and how much the output of the explanatory model would change if either parameter was randomly shuffled or data was randomly shuffled and the model was trained on the permutated labels. In their work, gradient-based methods are compared along with one perturbation-based method (65) and an edge detector (e.g., an algorithm that always illustrates the borders within an image). In the case in which parameters are randomized, the prediction model still preserves some capability to process information, using its structure as a prior. If a method is not sensitive to this permutation, then the explanation would not facilitate debugging the model, which is related to parameter learning. In the case in which labels are permuted, the relationship between the predictors and the labels based on the data-generation process is lost, and the model remembers each permutated example by “memorizing” it with over-fitting. If a method is insensitive to randomizing the labels, it implies the explanation generated does not depend on the data-generation process recorded by the model and, therefore, cannot explain the model from this perspective (60). In addition, Kindermans et al. (82) note that saliency maps can change their explanation when a transformation has no effect on how the model makes the decision, which suggests that these methods, although informative, still preside over robustness issues.

Aside from saliency maps, which are a local method, we may also visualize models trained on imaging data using a global metric to obtain a global interpretation. For example, in Ke et al. (31), the authors built an online EEG classifier for depression, in which they measured and visualized the information entropy (i.e., the amount of uncertainty contained in a particular random variable) of the activation matrix of each EEG channel. Because entropy correlates with the possible amount of information the model can utilize during learning, such a measure can serve as an indicator of importance for the model to make decisions.

### Interpreting Models With the Attention Mechanism

As mentioned previously, the attention mechanism was originally developed for applications in NLP. Language is the primary form of thought expression, carrying both contextual and syntactical information indispensable if one intends to learn about the mental state of another person. Indeed, in current psychiatric practice, assessments, diagnoses, and the majority of psychotherapies are carried out mostly by conversations or interviews in various forms as well as observations made and recorded by the therapist as clinical notes. Because the majority of such information is recorded in the form of text, NLP, the automation of text data analysis, is naturally an appealing component for psychiatry research.

In recent years, DNN-based NLP has undergone significant progress and has achieved state-of-the-art performance across many NLP tasks (27), which can be translated for use in psychiatric research. Recent progress began with the invention of distributed word representations, such as word2vec and GloVe (78, 83), which record co-occurrence information of words in vectorized forms. These techniques allow dimension reduction as well as a form of transferring prior knowledge of text into downstream models. A further breakthrough occurred with the invention of the attention mechanism (26), which attempts to build weights that reflect which part of the input is more important for decision making. Although originally invented for NLP, models with attention mechanism are not confined to applications with text data, but they can also fit any sequential and imaging data as well, making these models highly relevant to psychiatric research.

By design, it is natural to think that attention weights provide information on how decisions are being made. For example, through visualization, Clark et al. (73) systemically analyze attention layers of a landmark NLP model [“BERT” (27)] by first comparing behaviors across different layers as a trend and then focusing on behaviors of each attention head (e.g., a single set of attention values derived in an attention layer), during which they find certain attention heads were specialized in finding syntactic relations. The authors then probe the combined action of the attention heads within a single layer, and train supervised models based on labels of the location of the actual syntactic head of interest, using attention weights as predictors, showing the attention values are indeed predictive of the outcome. Last, they perform cluster analysis of all the attention heads in the model and show that heads in the same layer tend to be more proximate.

Interpreting through attention weights is not free of problems. For example, Jain et al. show that (74), (1) although perturbation- and gradient-based methods are consistent to a degree between their interpretations, attention-based methods yield interpretations that correlate more weakly to those two approaches; (2) changes in prediction outcome upon permutation of attention weights are modest in many cases; and (3) it is not impossible to find another set of attention weights that are quite different from the original while fixing other parts of the model, and the predictions are unchanged. These suggest that attention weights might not play the main “causal” role in making modeled decisions as it intuitively suggests.

Alternative approaches are created considering the issues. Ghaeini et al. (75) propose “attention saliency,” which, instead of looking at attention *per se*, visualizes a score defined by calculating the absolute value of the derivative of the model output with respect to the unnormalized attention weight and show that the attention saliency score provide more meaningful interpretation compared to vanilla attention weights on a natural language inference task. Instead of exploring attention weights, Aken et al. (76) takes advantage of the position-preserving nature of a BERT model in which the number of positions is constant across layers, and thus, the output of each layer can be perceived as a transformation of the input at the same position. They analyze the tokens produced by each attention layer from the BERT model, probe their properties with specific tasks, perform principle component analysis, and visualize clusters of token outputs at each layer.

## Discussion and Future Directions

In this paper, we review existing methods for DNN model interpretation that are suitable for most commonly collected types of data in contemporary psychiatric research. We also discuss a substantial proportion of research questions that can be addressed using ML approaches.

Indeed, the compositional nature and flexibility of DNNs carry both a blessing and a curse. Although DNNs bring out numerous breakthrough performances in a wide and growing variety of tasks, such complex systems are by nature more difficult to interpret thoroughly. In fact, our scientific body is just in the beginning stages of understanding some of their mathematical guarantees and why they works so well on many problems. For example, Poggio et al. (84) recently showed that the reason deep networks generalize well is partially explained by the fact that the gradient flow of a normalized network is intrinsically regularized and prove that approximation power of deep networks is superior to that of shallow networks under particular hierarchical compositional data structures. That being said, in real application, a sufficient interpretation for a particular instance does not necessarily involve a fully detailed understanding of all the mechanics. As summarized in this review, many of the interpretation methods utilize information that is most proximate to either the input or the output, and despite some mathematical properties yet to be met, these methods do yield interpretations that could serve a variety of purposes.

Making DNNs more interpretable is a fast-moving field on both the theoretical and engineering sides. To facilitate application and ease the burden of engineering for researchers, many methods discussed in this paper are published with their respective code libraries. At the moment of the preparation of this paper, libraries such as PyTorch Captum (85), tf-explain (86), and Google LIT (87) further simplify the process by providing compilations of off-the-shelf, easy-to-use implementations of the methods in common frameworks. It is noteworthy that, although the advent of new tools provides convenience, it is necessary to explicitly describe the underlying metric [i.e., what quantity is actually being visualized; for example, information entropy is measured in (31)] when applying these tools or visualizing for interpretations because different metrics provide measurements to different constructs, and their properties should be transparent and open to interrogation whenever necessary.

Many of the interpretability metrics are either a statistic or derived from a model apart from the model to be explained, and each may have its own issues in satisfying some desirable conditions (57–59, 68, 82, 88). Newer methods improve previous ones in accordance to some axioms, for example, integrated gradient improves on vanilla gradient in being sensitive when a specific neuron is saturated (68). New criteria are being iterated alongside new methods, and it is unsettled what would consist a standard set of desirable properties. This is also complicated by the fact that some of the properties are specific to the approach of interpretation. For example, “summation to delta” is specific to gradient-based methods. As new approaches are developed, new criteria specific to novel designs might be required. That said, it is desirable that at least a set of general properties, such as robustness as proposed by Alvarez-Melis (58)—e.g., interpretations for data sufficiently close should also be similar—should converge and be agreed upon.

It is well known that current DNN models by themselves are not entirely robust (i.e., sensitive to small perturbations in input data) (58), and their interpretation may be nonrobust to artifacts as well (62). As raised in Alvarez-Melis et al. (58), it is an open question if the method for interpretation should be required to be robust when the model itself is not. Indeed, it has not been directly investigated how desirable properties correlate between the model and its interpretation method. One potential direction for both modeling and interpretation is to capture more invariant structure (e.g., invariant to noise or certain transformations) in the data, for example, concept-based methods, i.e., approaches that either carry out predictions or can be interpreted by human-understandable concepts, such as ConceptSHAP (89, 90). Heuristically, finding or imposing invariant structures into the inner working of a model indeed should improve robustness. Nevertheless, if a model is expected to deliver supra-human performances, then it might not be always reasonable to expect the model to be fully interpretable using concepts that are readily understandable to humans.

An additional related issue arising from the nature of DNNs is the nonuniqueness of solutions, i.e., different sets of parameter estimations can be derived through repeatedly training the same model with the same data under slightly different conditions (e.g., initialization or hyper-parameters) as current optimization methods of DNNs can find different local minima across attempts (91). Selecting one particular solution undeterministically and automatically by the training algorithm among a set of possible solutions without a clear indication as to why that particular solution is chosen can by itself be seen as a violation of interpretability because it is then not clear why the algorithm would choose to do so. Therefore, an additional future direction to enhance DNN interpretability might be working toward model solutions that are more unique, possibly through ways such as improved data denoising, feature extraction, and representation learning that are more disentangled in order to smooth the landscape of the loss function.

One last noteworthy consideration is DNN interpretation in the context of few-shot of transfer learning (92), which seeks to enhance model performance under the constraint of small sample sizes by utilizing information not explicitly or directly related to the current task (i.e., injection of prior knowledge in various possible forms). Although not as pervasive as conventional methods in the meantime in psychiatric research, few-shot learning methods are in a rapid phase of development and is particularly of interest to the psychiatric research setting due to possible difficulties in case or label collection. At the time of the making of this paper, to the author's best knowledge, there have not yet been published articles that formally discuss the interpretation methods reviewed here in the context of few-shot learning. That said, heuristically, it is obvious that the meaning of derived interpretations can change based on the way few-shot learning is performed. In the case in which few-shot learning is done through data augmentation, the impact might be less likely to be significant, as the structure of the model, the initialization of parameters, the hypothesis space, and the amount of information used during model training are mostly identical to a non-few-shot setting. However, when few-shot learning is done with a change in some of the aforementioned conditions, the amount of information utilized during training can drastically decrease, and the pathway on which parameter values change over the course of training (i.e., gradients) can be shortened and altered. In these cases, the meaning given by the interpretation methods is then no longer “marginal” (i.e., relative to noninformative initiation) but conditional on the given known prior. In practice, depending on the architecture of the few-shot learning model, interpretation methods may have to be specifically tailored to the given model to perform well. As an example, in a recent work from Karlinsky et al. (93), the authors propose a few-shot learning model for image classification and show that vanilla GradCAM fails to provide visualization for some of the modeled examples, in which a back-projection map designed as an integral part of their model performed nicely.

In conclusion, with the current tools for interpretation, the “black box” of DNNs can have light shed on it and be inspected to an extent, and further improvements are constantly being made. After all, psychiatry itself is a very complex field, which implies mathematical models describing the patterns that have emerged in the field that would also be complicated and difficult to interpret. To solve this long-standing challenge, we must all be equipped to deal with and embrace the complexity when necessary. DNNs may act as a set of tools to help us discover patterns in psychiatric phenomenon that cannot be found otherwise. The various interpretation methods described above translate such discoveries into clinically meaningful and actionable findings. Alongside efforts to construct large and multidimensional data sets, a new wave of exciting exploration in psychiatric research awaits.

## Author Contributions

Y-hS is the sole author of this article and contributed to conception of idea, collection and summarizing materials, and the writing of the article.

## Conflict of Interest

The author declares that the research was conducted in the absence of any commercial or financial relationships that could be construed as a potential conflict of interest.
